# Who put the film in biofilm? The migration of a term from wastewater engineering to medicine and beyond

**DOI:** 10.1038/s41522-020-00183-3

**Published:** 2021-01-27

**Authors:** Hans-Curt Flemming, Philippe Baveye, Thomas R. Neu, Paul Stoodley, Ulrich Szewzyk, Jost Wingender, Stefan Wuertz

**Affiliations:** 1 Singapore Centre for Environmental Life Sciences Engineering (SCELSE), Nanyang Technological University Singapore, 60 Nanyang Drive, Singapore, 637551 Singapore; 2 University of Duisburg-Essen, Biofilm Centre, Universitätsstrasse 5, 45131 Essen, Germany; 3 Water Academy, Schloss-Strasse 40, 88045 Friedrichshafen, Germany; 4 Saint Loup Research Institute, 7 rue des chênes, 79600 Saint Loup Lamairé, France; 5 Department of River Ecology, Helmholtz Centre for Environmental Research–UFZ, Magdeburg, Germany; 6 Department of Microbial Infection and Immunity and the Department of Orthopaedics, The Ohio State University, Columbus, OH 43210 USA; 7 National Centre for Advanced Tribology at Southampton (nCATS), National Biofilm Innovation Centre (NBIC), Mechanical Engineering, University of Southampton, Southampton, S017 1BJ UK; 8 Technical University of Berlin, Department of Environmental Microbiology, Ernst-Reuter-Platz 1, D-10587 Berlin, Germany; 9 School of Civil and Environmental Engineering, Nanyang Technological University Singapore, 50 Nanyang Avenue, Singapore, 639798 Singapore

**Keywords:** Applied microbiology, Biofilms

## Abstract

Sessile microorganisms were described as early as the seventeenth century. However, the term biofilm arose only in the 1960s in wastewater treatment research and was adopted later in marine fouling and in medical and dental microbiology. The sessile mode of microbial life was gradually recognized to be predominant on Earth, and the term biofilm became established for the growth of microorganisms in aggregates, frequently associated with interfaces, although many, if not the majority, of them not being continuous “films” in the strict sense. In this sessile form of life, microorganisms live in close proximity in a matrix of extracellular polymeric substances (EPS). They share emerging properties, clearly distinct from solitary free floating planktonic microbial cells. Common characteristics include the formation of synergistic microconsortia, using the EPS matrix as an external digestion system, the formation of gradients and high biodiversity over microscopically small distances, resource capture and retention, facilitated gene exchange as well as intercellular communication, and enhanced tolerance to antimicrobials. Thus, biofilms belong to the class of collective systems in biology, like forests, beehives, or coral reefs, although the term film addresses only one form of the various manifestations of microbial aggregates. The uncertainty of this term is discussed, and it is acknowledged that it will not likely be replaced soon, but it is recommended to understand these communities in the broader sense of microbial aggregates.

## Motivation for the perspective

We recognize that there have been many previous discussions on the definition of the term “biofilm” but there has not been a review discussing the etymology of the term “biofilm”. By necessity we touch on both definitions and the historical development of biofilm microbiology which is establishing itself as a distinct discipline. Biofilm research is fundamental in nature revealing ever more complexities in these biological systems but is also highly applied, since biofilms impact many aspects of human life. Since the intended and perceived meanings of scientific terms may change as the discipline evolves we believe it is important to provide context behind the use of the term “biofilm”, how it was originally interpreted and how it is interpreted today. We believe that this is not only an academic exercise of historical interest but is important in helping to identify common ground for the exchange of ideas and techniques between biofilm researchers and others studying microbial communities, who might refer to their communities using different terminology. Arguably, the inherent interdisciplinary nature of biofilm research provides a centralized hub for those studying microbial communities from seemingly disparate fields ranging from industrial fouling to soil and sediment microbial ecology to wastewater engineering to chronic infections.

## Who put the film in biofilm?

The first report on microbial communities colonizing surfaces was published already in the seventeenth century where famously the aggregates of microbes in the “scurf” scraped from teeth and tongue were described^1^. The more widespread access to microscopes in the years to come made it possible for naturalists to study such microbial communities either in the field or in samples taken back to the lab. The objects of observation were usually plankton-containing aqueous samples, but also organisms colonizing surfaces such as the submersed leaves of plants or inert materials, e.g., rocks and sediments. The organisms examined and described were mostly animals, algae, and protists. The much smaller bacteria were hardly ever mentioned; only in communities with exceptionally large bacteria of distinct morphology, e.g., (phototrophic) sulfur bacteria or some iron bacteria, were such microorganisms described and even named. The terms used for these surface colonizing communities varied, e.g., “Aufwuchs” (German for “surface growth” or “overgrowth”)^2^, or epiphyton^3^.

Some of the earliest references to what we now call “biofilm” originated in wastewater treatment, marine fouling and dental microbiology. In these varied fields, the word “film” was commonly used to describe the biological layer that formed on solid surfaces and which, from macroscopic inspection or physical touch, appeared to be a continuous layer over rather large surfaces, particularly in biological wastewater treatment and marine fouling. In early microscopic studies the term “film” was introduced to account for the frequent observation that bacteria, and occasionally algae and protists, attached and multiplied on glass slides submerged in freshwater and marine environments. Microorganisms on slides exposed to lake water were shown to develop “in a fairly uniform film”^4^. On slides submerged in seawater “bacterial films” were identified and it was concluded that “bacteria and, to a lesser extent, other microorganisms are the primary film-formers on submerged glass slides” and that “such films favor the subsequent attachment of the larger and more inimical fouling organisms”, explaining the phenomenon of marine biofouling^5^. Subsequently, the sessile mode of microbial life was gradually recognized to be ubiquitous and predominant in natural as well as in engineered aquatic ecosystems as well as in commensal and pathogenic interactions with plants, animals, and humans^6–8^. From these observations the term “biofilm” became established as a general designation for sessile growth of microorganisms in diverse natural and built environments and industrial processes^8^.

From a technical point of view, “biological-”, “microbial-”, or “slime-films”^9–12^ were recognized to be beneficial such as in wastewater treatment, by supporting bacteria capable of biotransformation of multiple contaminants, but also detrimental to the shipping or power industry, by causing increased drag, heat transfer resistance, and corrosion. In wastewater treatment it was important to understand and model reactions mediated by such films in “biological film reactors” to optimize efficiency of these systems^12^. Although the “film” was utilized to break down waste, the rate at which waste contaminants moved into, and the broken down products moved out of, the film slowed as it grew thicker. As early as 1967 it was recognized that this “mass transfer limitation” was not only a function of film thickness, but also depended on the structure and heterogeneity of the film. Atkinson et al.^12^. noted in a hand drawn sketch that such films were not necessarily uniformly flat layers, but were heterogeneous and composed of microscopic features now referred to as “streamers”, “columns”, “mushrooms”, “clusters”, “microcolonies” and, more recently, “aggregates”^7,13–15^. Atkinson et al.^12^ concluded after modeling experimental data that the system could be adequately described using a one-dimensional model, assuming that the film was a continuous homogeneous layer. In engineering, the concept of “film theory” in fluid dynamics is a useful construct for understanding how the “no-slip condition” (i.e., the fact that even in a flowing system there is no flow immediately adjacent to the walls of the conduit) produces discrete boundary layers (as a useful idealized construct), which influence drag (the resistance of fluids to move past a solid surface or vice versa), heat and mass transfer between the bulk liquid in the main flow and the surface. Thus, it was a natural progression to make the simplifying assumption to incorporate the microbial layer as a continuous film into boundary layer film theory. The term “slime film” was later extended from biotransformation to fouling drag in industrial process and production plant pipelines^16^.

The first incidence of the migration from using the term “biological film” to “biofilm” that we are aware of occurred in 1975 in a paper describing processes in a “microbial film trickling filter” used for wastewater purification^17^, as a natural extension of characterizing and predicting the effect of microbial fouling on heat transfer and drag. First, Characklis^16,18^, Marshall^19^, Geesey et al.^20^ and Costerton et al.^6^, all pioneers in the field, studied films at interfaces in a variety of environmental habitats. Several studies looked at controlling of such films with antimicrobials and made the discovery that microbes in biofilms were much more difficult to kill than single planktonic cells^21,22^.

This commonality of microbial surface attachment and their recalcitrance to chemical treatment unified evolving concepts to describe biofilms in industrial systems to extend to those causing medical infections. However, medically relevant biofilms were constrained by the scale of human anatomy to much smaller areas. Observation of clinical samples, first with scanning and transmission electron microscopy and later with confocal laser scanning microscopy, has revealed that in many cases such biofilms represent discrete aggregates of microbial cells rather than continuous films^23^. Non-attached cell aggregates of bacterial pathogens have been observed in chronic infections, where they are localized in the lumen of organs or within tissues, e.g., in the lungs of cystic fibrosis patients and in non-healing wounds. As these medically relevant aggregates show features resembling those of surface-attached biofilms such as EPS production, slow growth rate, and tolerance towards antibiotics and host immune cells, they have been included in the biofilm definition^24^. Many in the medical community now associate biofilms with a number of unique properties and the clinically relevant consequences in terms of prophylaxis and treatment strategies—and after several decades of growing awareness of their existence and an understanding of how these localized microbial communities may explain the natural history and recalcitrance to treatment of many chronic localized infections, the term is becoming widely accepted by practitioners^8,25^.

When discussing the terms film and/or biofilm, the surface to which the film/biofilm is attached to, or more broadly, the interface with which it is associated, immediately comes into focus. Although Marshall, in his pioneering work “Interfaces in Microbial Ecology”^26^ did not use the term biofilm, he was very much aware of the significance of interfaces for the development of microbial communities. Consequently, he included many references related to films, flocs, aggregates, and microlayers. Nevertheless, it took several years for the first books with the term “biofilm” in the title to be published^27,28^. Thereby, the topic slowly started to establish itself as a research field of its own. Currently, it is accepted that biofilms are manifestations of microbial life not only growing on surfaces but developing at any solid-liquid, liquid–liquid, liquid–gas, and solid–gas interface^26,27^.

## Biofilm—a term ready for the next leap

In the last few decades, the term biofilm has grown in usage while still remaining somewhat vague to non-specialists. It suggests the presence of a surface, on which a film of microorganisms is spread—with cells colonizing in patchy, thin, or multilayered patterns. Accordingly, a number of early conceptual and mathematical models were proposed for the diverse appearances of microbial biofilms on surfaces, for example, the heterogeneous mosaic biofilm model^29^, the penetrated water-channel mushroom-like biofilm model^30^, and the dense confluent biofilm model^31,32^. In the heterogeneous mosaic biofilm model based on the analysis of biofilms in oligotrophic drinking water systems the biofilm usually consists of a thin (≈5 µm) basal layer of microorganisms with tall stacks (up to 100 µm) of microcolonies rising from the surfaces. In the water-channel model, microcolonies form mushroom-like structures attached by stalks of EPS and microorganisms which may merge into each other and are penetrated by water channels^30^. This model was proposed on the basis of observations of laboratory biofilms of *Pseudomonas aeruginosa* grown in flow cells under relatively high nutrient conditions, as well as other strains or mixed cultures; however, these laboratory-grown biofilm structures are not representative for many environmental biofilms. The dense confluent biofilm model has been described for medically important biofilms such as oral biofilms (dental plaque)^32^ as well as for aquatic environments^31^. Observations of natural and laboratory-grown biofilms as well as computer modeling of biofilm growth suggested that biofilm structure may be largely determined by the prevailing substrate concentration^33^ and hydrodynamic conditions^34^. In natural and industrial ecosystem biofilms grazing by protozoa can also influence structure^35^. There may be a continuum between these types of biofilms as, for example, an increase in nutrient availability may cause a transition from sparsely colonized surfaces with heterogeneous biofilms to confluent and compact biofilms that correspond to the initial notion of biofilms as surface-covering biological layers.

## Biofilms shaped by extracellular polymeric substances (EPS)

Research on biofilms living at diverse types of interfaces reveals commonality in the way in which these microorganisms live, often embedded in a matrix of EPS^36,37^. This matrix is self-secreted and protective, surrounding and immobilizing microbial cells within a biofilm so that they can establish those stable spatial interactions not possible in well-mixed planktonic populations. The EPS comprise polysaccharides, proteins, lipids, and nucleic acids—most of them highly hydrated, but also containing water-insoluble compounds such as cellulose or amyloids—all of which can form the scaffold of the matrix alone or through interactions of these polymers. The EPS also contain bacterial derived refractory compounds^23^ as well as molecules and particles sorbed from the local environment (quartz, basalt, clay minerals). Importantly, the EPS are responsible for capturing inorganic constituents whose deposition is clearly distinct from the pure inorganic chemical deposition of scaling. The matrix exists in a continuum of physical states ranging from dissolved to dense gels and sometimes biogeomineral precipitates, e.g., iron and manganese oxides and calcium carbonate^38,39^. One issue in recognizing these outwardly appearing inorganic fouling deposits as biofilms is that the biomass may be an extremely small fraction of the deposit, even though the associated bacteria may initiate or accelerate the inorganic deposition^40^. It is clear that the EPS largely determine the mechanical properties of biofilms, which can range from rigid solids, to viscoelastic permeable solids^41^ to highly viscous liquids^42^, depending on the type of biofilm and the manner in which the biofilm is mechanically stressed^43–45^. The matrix enhances biogeochemical gradients in the micrometer scale resulting from microbial activities^46^. The EPS are responsible for the “gluing” interactions between interfaces and cells as well as with dissolved and particulate compounds. The EPS provide balance between structural stability and a degree of compliance that allows the development of structured microbial communities with emergent properties that are clearly distinct from planktonic single cells^15^. Key to these properties is the close proximity of the cells over extended periods of time, facilitated by long retention times in juxtaposition within the EPS matrix. Some of the properties emerging from this situation are listed here^15,23^: These include:

- Retention of extracellular enzymes which provides enzymatic products closely to the cells, representing an extracellular digestion system
- Formation of localized gradients in pH, dissolved oxygen, redox potential, nutrient and metabolic product concentration and, as a consequence, microenvironments for habitat diversity.
- Resource capture and retention of cellular products and debris which can be used as a reservoir for biological transformations.
- Water retention, protecting against dehydration.
- Allowing for long-term development of synergistic consortia between different species.
- Enhanced tolerance against antibiotics, biocides, and other chemical or physical antimicrobial agents.
- Facilitated gene exchange and recycling of nucleic acids, representing a genetic pool for horizontal gene transfer.
- Enhanced intercellular communication and collective behavior.
- Continuous regeneration by competition in response to stressful and changing environmental conditions.
- Habitat formation.

Biofilms as a characterization of microbial communities is not restricted to microbial films on surfaces but applies to multicellular microbial aggregates in general. They comprise a wide range of manifestations including endolithic microbial populations, flocs, pellicles, slimes, zooglea, microbial mats, microorganisms in soils, sediments and aerosols, bacterial neuston in the aquatic surface microlayer, marine and lake snow, biofilms on leaves and plant roots, activated and granular sludge, biofilters in drinking and wastewater purification, aggregates in biofouling and microbially influenced corrosion and weathering, and many more^47^. These phenomena have been characterized by Moshynets and Spiers^48^ as a “continuum of aggregations”. In spite of their vast phenomenological diversity, they share common properties, unifying them in a class of complex collective ecosystems, such as forests, coral reefs, and social insect communities^15^. Similar to such communities, microbial biofilms act as “protective and internally homeostatic fortresses at a scale much larger than the living organisms who built them” and fit into the concept of “extended organisms”^49,50^.

It is obvious that the term “film” does not fit every aggregate in this huge spectrum. Understandably, the respective scientific communities addressing sectors of this spectrum were hesitant about renaming accepted terms such “dental plaque”, “activated sludge”, “microbial mats”, “desert varnish”, “soil aggregates”^51^ or others as “films”, since these terms are already well established in their respective disciplines. The term biofilm was uncommon in literature on soils, where the term “biofilm” traditionally did not appear until recently^52^, because researchers had failed to observe anything resembling films in these systems^53,54^. The latter authors preferred to refer instead to “groups of cells” located in specific microenvironments. In this discipline, as in others before, the push by some to adopt the “biofilm” terminology has led to occasionally heated discussions among “biofilm” researchers and those not happy questioning their field of research requiring a new label^54,55^.

The jury is still out on whether there can be an acceptable alternative to the term “biofilm” that would better describe all the manifestations of the “continuum of aggregations”^48^. Incorporation of the term “aggregate“ may be useful since it already appears in the IUPAC definition of biofilms^14^: “Biofilms are aggregates of microorganisms in which cells are frequently embedded in a self-produced matrix of extracellular polymeric substances (EPS) that are adherent to each other and/or an interface”. In this slightly amended definition, we have substituted the original word “surface” with “interface” to meet the expanded view of biofilm phenomena (see ref. ^47^. It includes the vast variety of microorganisms, but also other biotic and/or abiotic components included in or held together by the EPS matrix. We point out that aggregate formation and growth can be through microbial growth, collision–adhesion interactions or a combination of both. One downside of the term “aggregate” is that, albeit more appropriate, it is definitely less catchy than”biofilm”. Another issue is that in some fields (for example, in geology, soil science, or in materials science) the term is already used to describe collections of abiotic particulate matter, not involving microorganisms at all, so that using aggregates there might lead to confusion. An alternative to “biofilm” suggested by Neu and Lawrence^56^ is “biofilm system” which includes both “bio-films” and “bio-aggregates”. In the context of soils, use of the term “bio-cluster” was suggested, acknowledging that a key feature of the associations of cells and extracellular polymers found in terrestrial environments is that clay particles are very often adsorbed to them as a kind of sheath.

It is interesting to perceive that a large proportion of microbial community research is not directed to single, planktonic cells but to biofilms in the broad sense of our definition (e.g., in sediments^57^, soils^58^, wastewater^59^, or the human gut^60^, to name a few). Usually, the biofilm aspects in terms of interactions among the members of the communities are not considered.

Although it is unlikely that the term “biofilm” will be replaced any time soon, we hope that our perspective will encourage circumspection before adopting the term in fields where historically its use has not been routine, and where there are other options. As a broader take-home message, it is worth remembering that sensu stricto, the term biofilm can serve only a section of the wide spectrum of manifestations of cell–EPS aggregates on earth^47^.
